# Exploration of the value of concurrent chemotherapy for T2N1 nasopharyngeal carcinoma under intensity modulated radiotherapy mode

**DOI:** 10.3389/fonc.2024.1424804

**Published:** 2024-11-18

**Authors:** Kai Liao, Jian Zhang, Wenze Qiu, Ronghui Zheng

**Affiliations:** Department of Radiotherapy, Guangzhou Institute of Cancer Research, the Affiliated Cancer Hospital, Guangzhou Medical University, Guangzhou, China

**Keywords:** nasopharyngeal carcinoma, T2N1, intensity-modulated radiation therapy (IMRT), concurrent chemoradiotherapy (CCRT), therapeutic outcome, side effect

## Abstract

**Problem:**

In the era of intensity-modulated radiation therapy (IMRT), the status of concurrent chemoradiotherapy(CCRT) for stage II nasopharyngeal carcinoma(NPC), particularly for patients in T2N1 subtype, remains controversial nowadays.

**Aim:**

This study exclusively aims to explore the value of concurrent chemotherapy in the treatment of T2N1 NPC under IMRT mode.

**Methods:**

A retrospective analysis was conducted on 218 cases of T2N1 NPC patients treated at our hospital from January 2015 to December 2020, comprising 75 cases treated with IMRT and 143 cases treated with CCRT. The study compared therapeutic outcomes and side effects between the two groups.

**Results:**

The 5-year progression-free survival (PFS), overall survival (OS), locoregional relapse-free survival (LRRFS) and,distant metastasis-free survival (DMFS) estimated by the K-M method for the IMRT vs. CCRT groups were 86.1% vs. 85.1%,89.3% vs. 87.9%, 95.9% vs. 94.9%,and 90.2% vs. 89.1%, respectively, with no statistically significant differences (Log-rank *P*>0.05 for all comparisons). Cox regression analysis identified Epstein-Barr virus (EBV) DNA copy level (≥1000 vs. <1000 copies/ml)(the cutoff value was determined through the ROC curve), lymph node necrosis (yes vs. no) and extra-nodal extension (yes vs. no) as independent prognostic factors for PFS(*P*<0.05 for all comparisons). Subgroup analysis indicated an interaction effect between lymph node necrosis (yes vs. no) and treatment modality (IMRT vs. CCRT) regarding PFS (*P* for interaction<0.05). In the subgroup with lymph node necrosis, IMRT compared to CCRT had a poorer prognosis (HR: 1.85,95% CI: 1.02-3.50). CCRT was noted to increase acute hematological, gastrointestinal and other toxicities.

**Conclusions:**

This study provides a reference for clinical treatment decisions in T2N1 NPC. For the entire population of T2N1 NPC, the therapeutic effects of IMRT and CCRT are comparable, with increased acute toxicities in the latter. However, for patients with EBV-DNA copy level ≥1000 copies/ml, lymph node necrosis and extra-nodal extension, CCRT may be considered as appropriate. Particularly, patients with lymph node necrosis may be potential beneficiaries for CCRT.

## Introduction

Nasopharyngeal carcinoma (NPC),a malignant tumor arising from the nasopharyngeal mucosa, is particularly prevalent in southern China. The International Agency for Research on Cancer estimated that there were approximately 129,000 patients worldwide in 2018, and 47.7% of those occurred in China (1). Given its unique anatomical location and tumor biological characteristics,radiotherapy has become the primary treatment modality,with intensity-modulated radiation therapy (IMRT) being the most mainstream radiotherapy technology currently employed (1). In the era of IMRT,Stage I NPC can be cured with radiotherapy alone,and platinum-based concurrent chemoradiotherapy (CCRT) has been firmly established for locally advanced NPC in stages III-IVa,but the role of concurrent chemotherapy in stage II NPC,particularly for patients in T2N1 subtype,remains controversial (2).

A phase III single-center randomized controlled study published in 2011, which utilized 2-dimensional radiotherapy technology, recommended CCRT for stage II NPC. It demonstrated that CCRT improved therapeutic outcomes compared to radiotherapy alone (3). However, the benefit of concurrent chemotherapy may be due to its radiosensitizing effect, potentially compensating for the dosimetric insufficiencies in target coverage associated with traditional two-dimensional radiotherapy technology (4). With the advent of IMRT, which offers significant dosimetric advantages, both local control rates and survival rates for NPC have seen markedly improved. Particularly for stage II NPC, which responds well to IMRT alone, the additional benefit of concurrent chemotherapy in enhancing efficacy may be minimal (5). Whether concurrent chemotherapy is truly necessary for stage II patients with IMRT has garnered more attention. So far, two prospective randomized controlled clinical trials(RCTs), published separately in 2020 (6) and 2022 (7), have been conducted to explore the value of concurrent chemotherapy in stage II NPC, but both of them showed that concurrent chemoradiotherapy compared with IMRT alone did not improve survival rates but increased side effects for stage II patients.

However, stage II NPC exhibits significant heterogeneity, which is divided into three subtypes: T2N0, T1N1, and T2N1 based on the 8th edition of the UICC staging system,with distant metastasis remaining the predominant pattern of treatment failure (8). The aforementioned 2020 phase II clinical study (6) included 84 stage II NPC patients, with 41 in the CCRT group and 43 in the IMRT group. Out of five patients with distant metastasis, four had the T2N1 subtype. This suggested that T2N1 had the highest risk of metastasis and the poorest prognosis among the three subtypes of stage II NPC. However, due to the small sample size, further multivariate and subgroup analyses could not be performed to clarify the value of concurrent chemotherapy in different subtypes. The authors suggested that it would be important to distinguish patients with a higher risk of distant metastasis from general stage II patients to provide them with a more tailored treatment strategy. At the same time, multiple retrospective studies have reported that T2N1 patients had a significantly poorer prognosis than the other two subtypes, presenting the highest risk of treatment failure (9–11). The 2022 phase III clinical study (7), the other of the two RCTs,included low-risk stage II and T3N0 patients,with low-risk criteria defined as all lymph nodes<3 cm, no lymph node metastasis in the level IV/Vb, no ENE and EBV-DNA copy level<4000 copies/ml. The results showed that the 3-year failure-free survival rate of IMRT was non-inferior to CCRT, with similar outcomes observed in the T2 and N1 subgroups. However, the study focused on a highly selective population,excluding many high-risk factors. In the real world, there are more T2N1 patients with high-risk factors, and it remains unclear whether it is safe to omit concurrent chemotherapy. In summary, for the T2N1 subtype, which has an unmet need in terms of treatment, the question of whether concurrent chemotherapy can be entirely omitted and whether the intensity of IMRT alone is sufficient requires further investigation.

Hence, this study is the first to exclusively focus on the T2N1 subtype, which has the most unfavorable prognosis among stage II NPC patients, aiming to thoroughly explore the value of concurrent chemotherapy in addition to IMRT, rather than considering all stage II patients as a homogeneous group.

In our study, we focused on the treament of T2N1 NPC and have made the following primary contributions:

Contribution 1: We focused exclusively on the T2N1 NPC which has an unmet need in terms of treatment, for the first time, rather than encompassing all stage II patients, to thoroughly explore the value of concurrent chemotherapy on the basis of IMRT.

Contribution 2: Through analysis, we uncovered that for T2N1 NPC with EBV-DNA copy level ≥1000 copies/ml, lymph node necrosis and ENE, CCRT might be considered as appropriate. In particular, patients with lymph node necrosis might be potential beneficiaries of CCRT.

Contribution 3: We contributed valuable insights into treatment decision-making for patients with this specific type of NPC.

The structure of this paper is as follows: Section 2 reviews the inclusion criteria and research methodology of the study; Section 3 describes the study results in detail; and Section 4 discusses the significance and causes of these results, limitations of the study, suggestions for future research and conclusion of the study.

## Materials and methods

### Patients

**Figure 1:**
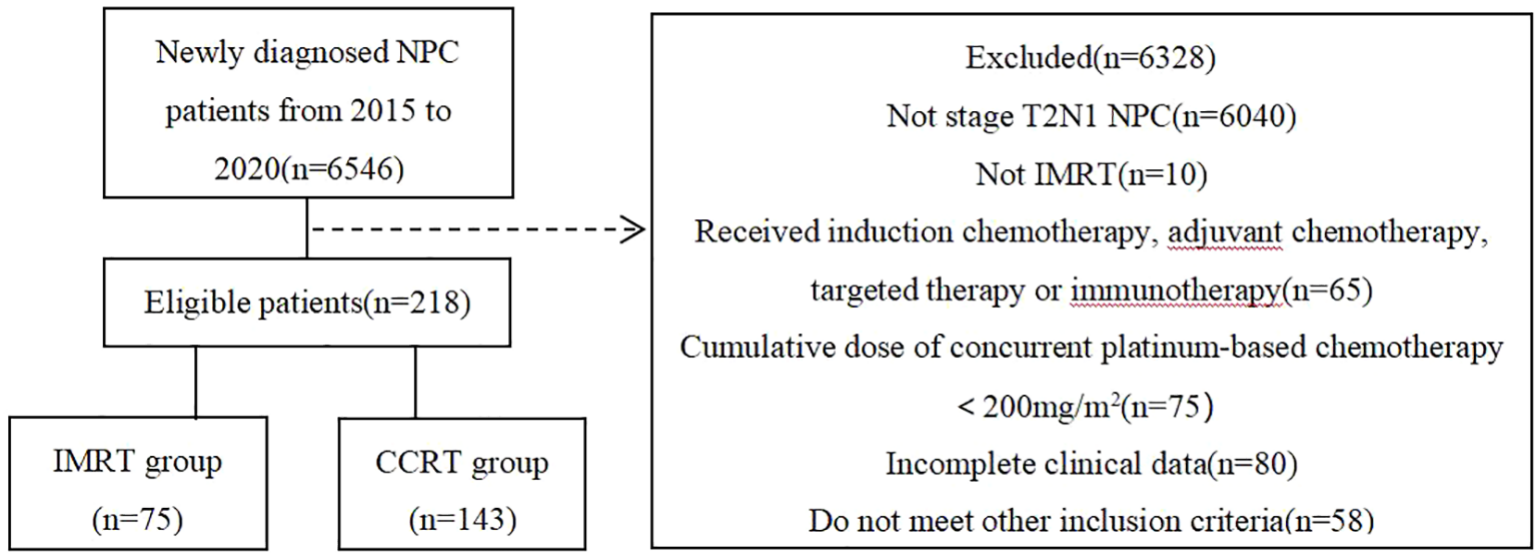
Flow chart of patients inclusion.

A retrospective analysis was performed on NPC patients consecutively admitted to Guangzhou Institute of Cancer Research, the Affiliated Cancer Hospital, Guangzhou Medical University, China, from January 2015 to December 2020. Inclusion criteria were as follows (1): patients re-staged as T2N1M0 according to the UICC/AJCC 8th edition; (2) ages between 18-70 years old; (3) received either IMRT alone or platinum-based CCRT, without induction chemotherapy, adjuvant chemotherapy,targeted therapy,or immunotherapy; (4) achieved a radical radiation dose of at least 68-70Gy; (5) the cumulative dose of concurrent platinum-based chemotherapy was at least 200mg/m^2^; (6) complete clinical data including pre-treatment nasopharyngeal and neck enhanced MR and plasma EB virus(EBV) DNA copy level, etc.; (7) no history of other malignant tumors. Finally, a total of 218 patients were included in the analysis, divided into two groups based on the presence or absence of concurrent chemotherapy, with 75 cases in the IMRT group and 143 cases in the CCRT group. The flow chart of patient inclusion is shown in **Figure 1**.

### Baseline data acquisition

All patients were re-staged according to the UICC/AJCC 8th edition. We collected patients’ general demographic information (such as gender, age, PS score, smoking history, family history of NPC,etc.), information related to the primary lesion (such as GTVnx volume,etc.), information related to regional lymph nodes (such as maximum diameter of lymph nodes, lymph node necrosis, extracapsular extension, metastatic lymph node regions,etc.), as well as serum tumor markers (such as EBV-DNA, lactate dehydrogenase(LDH),etc.), and so on. The GTVnx volume was calculated using the treatment planning system,including the primary lesion+retropharyngeal lymph nodes(LNs). The maximum diameter of cervical lymph nodes was determined from MR cross-sectional images, and all lymph nodes were assessed for necrosis and extra-nodal extension(ENE). Necrosis was diagnosed based on central T1 hypointensity, T2 hyperintensity,and rim enhancement on lymph nodes (12). ENE was diagnosed based on blurred lymph node boundaries, irregular enhancement of the capsule,fusion or infiltration into adjacent fat or muscle (13).

### Treatment

All patients received radical IMRT. The gross tumor volume of the nasopharynx (GTVnx) included the nasopharyngeal tumor and positive retropharyngeal lymph nodes as identified by clinical and imaging examinations. The gross tumor volume of the lymph nodes (GTVnd) included positive cervical lymph nodes detected by clinical and imaging examination. The first clinical target volume (CTV1) was extended 0.5cm from the GTVnx in all directions,which included all nasopharyngeal mucosa and 0.5cm below it. The second clinical target volume (CTV2) was expanded 0.5cm from the CTV1 in all directions (following the principle of distance+structure,and the expansion distance was determined according to the characteristics of the adjacent tissue), as well as the lymph node drainage area that needed to be irradiated prophylactically. The planning target volumes for the nasopharynx (PTVnx), lymph nodes (PTVnd), the first planning target volume (PTV1), and the second planning target volume (PTV2) were formed by expanding GTVnx, GTVnd, CTV1, and CTV2 0.5cm in all directions, respectively. The prescribed doses were: PTVnx/PTVnd 68-70Gy/32-33F, PTV1 60-62Gy/32-33F,PTV2 54-56Gy/32-33F. The concurrent chemotherapy regimen was cisplatin or nedaplatin,administered weekly at 40mg/m^2^ for a total of 5-6 cycles,or every three weeks at 80-100mg/m^2^ for a total of 2-3 cycles.

### Follow-up and end points

Patients were followed up every three months in the first two years after treatment, every six months in the 3rd to 5th year, and annually thereafter. Follow-up included physical examination, complete blood count, blood biochemistry, EBV-DNA, nasoendoscopy, nasopharyngeal + neck MRI, chest X-ray or CT, upper abdominal ultrasonography or CT, bone scan, and PET/CT if necessary. The primary endpoint was progression-free survival (PFS), defined as the time interval from the start of treatment to recurrence, metastasis, or death. Secondary endpoints included overall survival (OS), defined as the time interval from the start of treatment to death from any cause;locoregional relapse-free survival (LRRFS), defined as the time interval from the start of treatment to the first occurrence of local/regional recurrence; distant metastasis-free survival (DMFS), defined as the time interval from the start of treatment to the first occurrence of distant metastasis; and side effects. Side effects were evaluated using the NCI-CTC AE 4.0 criteria.

### Statistical methods

SPSS 22.0 statistical software (IBM Corp.,Armonk,NY,USA) was used. Quantitative data were transformed into categorical data through median or cut-off values,where the optimal cut-off values for EBV-DNA copy level and GTVnx volume regarding PFS were obtained through ROC curve analysis. Categorical data were described using frequency and composition ratio, and comparisons were made using the chi-square test or Fisher’s exact test. The Kaplan-Meier method was used to estimate survival rates, and the Log-rank test was applied for between-group comparisons. Hazard ratios (HR) and their 95% confidence intervals (CI) were calculated using the Cox proportional hazards regression model. Univariate analysis was performed to identify prognostic factors for PFS. Treatment modality (IMRT vs. CCRT) and factors with P<0.05 in univariate analysis were further included in multivariate analysis to determine independent prognostic factors,using the Forward(Wald) procedure for variable selection. Subgroup analysis was performed by including interaction terms (product terms) into the COX regression, to calculate the P value for interaction,and forest plots were plotted using GraphPad Prism 9.5.0 software. A two-sided test was used, with *P*<0.05 indicating statistical significance.

## Results

### Clinical characteristics

**Figure 2:**
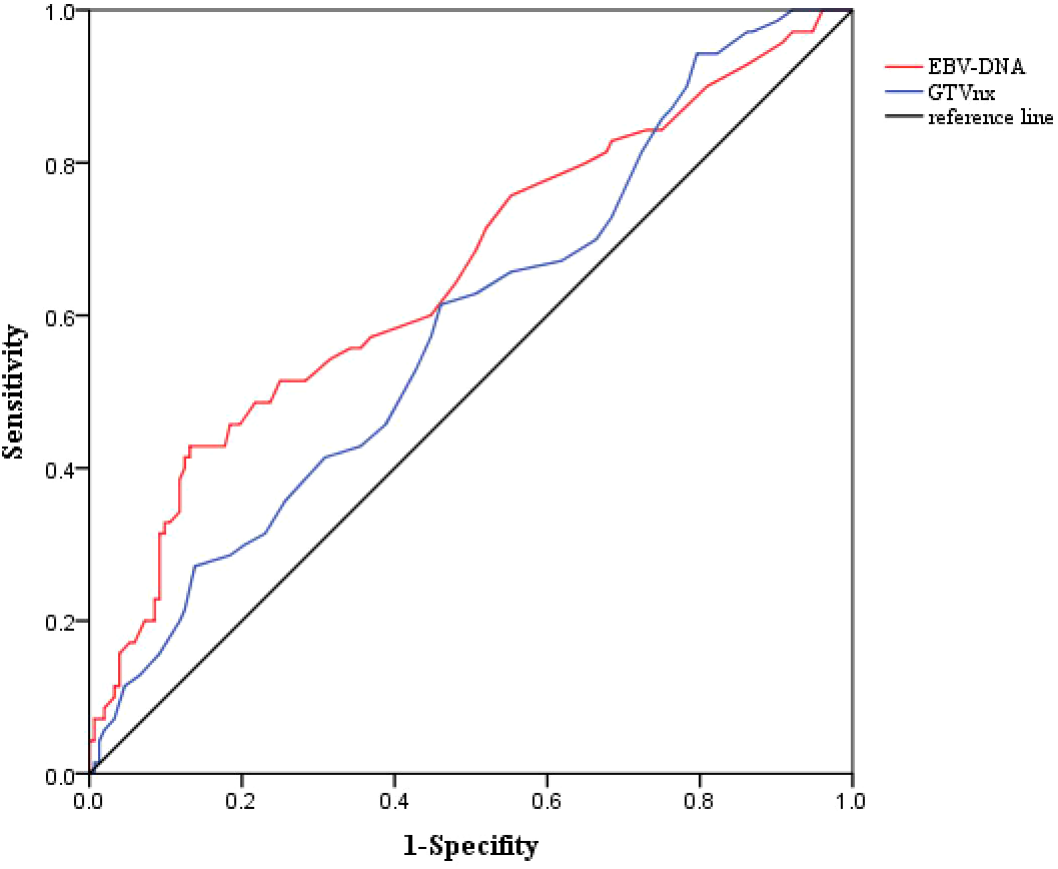
ROC curves of EBV-DNA and GTVnx for PFS.

The clinical characteristics of the patients are shown in **Table 1**. A total of 218 patients were included, with 75 in the IMRT group and 143 in the CCRT group. All patients were histologically confirmed as undifferentiated non-keratinizing carcinoma(WHO type III). The ratio of male to female patients in the total population was 2.69:1. The median age was 48 years (range:20-69 years). Approximately 30.3% of patients had a history of smoking, and 5.5% had a family history of NPC. The median pre-treatment plasma EBV-DNA copy level was 650 copies/ml,with a range of 0 to 10,500 copies/ml. Based on ROC curve analysis, the optimal cutoff value for PFS was determined to be 1000 copies/ml (sensitivity:0.514,specificity:0.750,AUC=0.655 (0.575-0.735),P<0.001) when the Youden index (sensitivity+specificity-1) was maximized,and this cutoff was used to convert EBV-DNA into a categorical variable (as shown in **Figure 2**). Nearly 5.0% of the patients had pre-treatment LDH levels equal to or exceeding the normal upper limit(245U/L). The median GTVnx volume was 18.0 ml (range:10.5-65.0 ml),and the optimal cut-off value for PFS determined by ROC curve analysis was 22ml(sensitivity:0.614, specificity:0.539, AUC=0.587 (0.507-0.666),P=0.038) (as shown in **Figure 2**). The proportion of cervical lymph nodes with a maximum diameter ≥3 cm and lymph node necrosis were 20.6% both. The proportion with ENE was 15.6%, and the proportion with lymph node metastasis in the level III was 31.2%. There were no statistically significant differences in the baseline clinical characteristics between the IMRT and CCRT groups (*P* all > 0.05).

**Table 1:** Comparison of baseline clinical characteristics between the two groups of patients.

Item	IMRT[cases (%)]	CCRT[cases (%)]	*χ^2^*	*P*
Total	75 (34.4)	143 (65.6)		
Age (years)				
<48	33 (44.0)	75 (52.4)	1.40	0.236
≥48	42 (56.0)	68 (47.6)		
Gender				
Male	55 (73.3)	104 (72.7)	0.01	0.924
Female	20 (26.7)	39 (27.3)		
PS score				
0	3 (4.0)	15 (10.5)	2.74	0.098
1	72 (96.0)	128 (89.5)		
History of smoking				
Yes	18 (24.0)	48 (33.6)	2.13	0.144
No	57 (76.0)	95 (66.4)		
Family history of NPC				
Yes	7 (9.3)	5 (3.5)	3.22	0.073
No	68 (90.7)	138 (96.5)		
EBV-DNA (copies/ml)				
<1000	48 (64.0)	85 (59.4)	0.43	0.512
≥1000	27 (36.0)	58 (40.6)		
LDH (U/L)				
<245	70 (93.3)	137 (95.8)	0.63	0.429
≥245	5 (6.7)	6 (4.2)		
GTVnx volume (ml)				
<22	42 (56.0)	88 (61.5)	0.63	0.429
≥22	33 (44.0)	55 (38.5)		
Maximum diameter of LNs (cm)				
<3	65 (86.7)	108 (75.5)	3.73	0.054
≥3	10 (13.3)	35 (24.5)		
Necrosis of LNs				
Yes	13 (17.3)	32 (22.4)	0.76	0.382
No	62 (82.7)	111 (77.6)		
ENE				
Yes	11 (14.7)	23 (16.1)	0.08	0.784
No	64 (85.3)	120 (83.9)		
LNs located in level III				
Yes	18 (24.0)	50 (35.0)	2.76	0.097
No	57 (76.0)	93 (65.0)		

### Survival analysis

**Figure 3:**
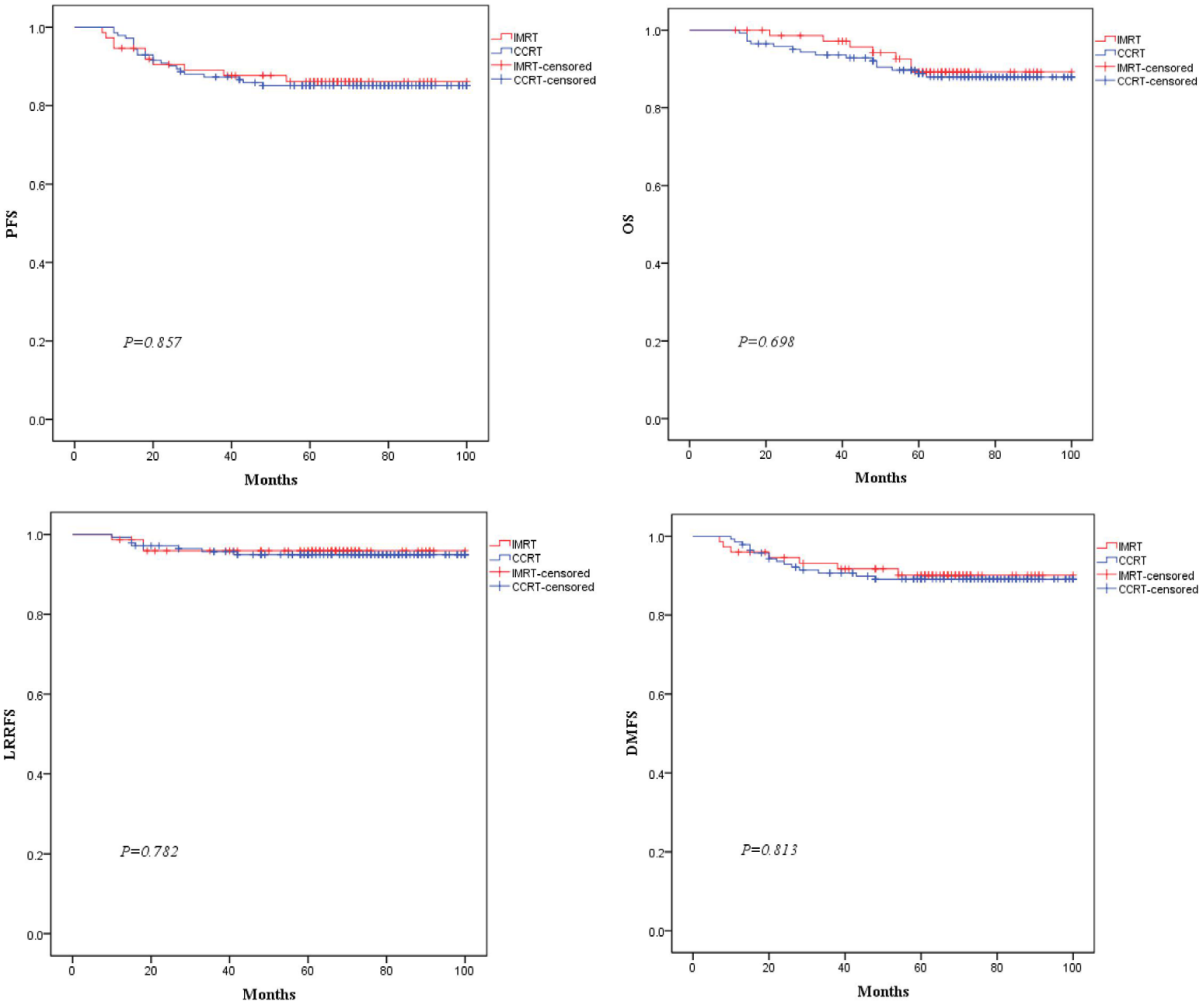
Comparison of Kaplan-Meier survival curves for PFS, OS, LRRFS, and DMFS between the two groups of patients.

The median follow-up time was 62 months (range:12-100 months). In the total population,there were 31,23,10, and 22 endpoint events for PFS,OS,LRRFS,and DMFS, respectively. As shown in **Figure 3**, the 5-year PFS, OS, LRRFS and DMFS rates estimated by the K-M method for the IMRT vs. CCRT groups were 86.1% vs. 85.1% (*χ2 =* 0.03, *P*=0.857), 89.3% vs. 87.9% (*χ2 =* 0.15, *P*=0.698), 95.9% vs. 94.9% (*χ2 =* 0.08, *P*=0.782), and 90.2% vs. 89.1% (*χ2 =* 0.06, *P*=0.813), respectively, with no statistically significant differences.

### Prognostic factor analysis for PFS

In this study,the number of endpoint events for OS, LRRFS and DMFS was relatively small. According to the 5-10 Events Per Variable(EPV) principle for sample size estimation in multivariate analysis using the Cox proportional hazards regression model (14), it is generally required that the effective sample size, i.e., the number of endpoint events, should be no less than 5-10 times the number of covariates included in the model,so performing Cox regression analysis on OS, LRRFS and DMFS with insufficient effective sample size may lead to unstable models and erroneous conclusions. Since this study focused on PFS as the primary endpoint, risk factor analysis was only performed for PFS. As shown in **Table 2**, univariate analysis indicated that EBV-DNA copy level (≥1000 vs.<1000 copies/ml), GTVnx volume (≥22 vs.<22 ml), maximum diameter of cervical lymph nodes (≥3 vs.<3 cm), lymph node necrosis(yes vs. no) and ENE(yes vs. no) were prognostic factors for PFS (*P*<0.05 for all comparisons). When treatment modality (IMRT vs. CCRT) and the above five factors were included in multivariate analysis,three factors remained in the model: EBV-DNA copy level, lymph node necrosis and ENE (*P*<0.05 for all comparisons), which were identified as independent prognostic factors for PFS. Therefore, for T2N1 patients with EBV-DNA≥1000 copies/ml, lymph node necrosis or ENE, the risk of failure was increased, and CCRT might be considered as appropriate.

**Table 2:** Univariate and multivariate analysis of PFS.

Variable	Univariate analysis of PFS		Multivariate analysis of PFS	
	HR (95%CI)	*P*	HR (95%CI)	*P*
Age (years) (<48 vs. ≥48)	0.94 (0.56-1.55)	0.809		
Gender (Male vs. Female)	0.93 (0.60-1.42)	0.732		
PS score (0 vs.1)	1.01 (0.43-2.32)	0.986		
History of smoking (Yes vs. No)	1.37 (0.95-1.99)	0.098		
Family history of NPC (Yes vs. No)	1.31 (0.65-2.60)	0.438		
EBV-DNA (copies/ml) (<1000 vs. ≥1000)	0.61 (0.38-0.93)	0.024	0.63 (0.41-0.99)	0.046
LDH (U/L) (<245 vs. ≥245)	0.83 (0.52-1.32)	0.418		
GTVnx volume (ml) (<22 vs. ≥22)	0.58 (0.35-0.98)	0.041		
Maximum diameter of LNs (cm) (<3 vs. ≥3)	0.55 (0.30-0.99)	0.045		
Necrosis of LNs (Yes vs. No)	2.45 (1.64-3.51)	<0.001	2.52 (1.73-3.61)	<0.001
ENE (Yes vs. No)	2.24 (1.31-3.65)	0.002	2.30 (1.37-3.70)	<0.001
LNs located in level III (Yes vs. No)	1.39 (0.68-2.81)	0.366		
Treatment modality (IMRT vs. CCRT)	0.93 (0.44-1.98)	0.857		

### Subgroup analysis

**Figure 4:**
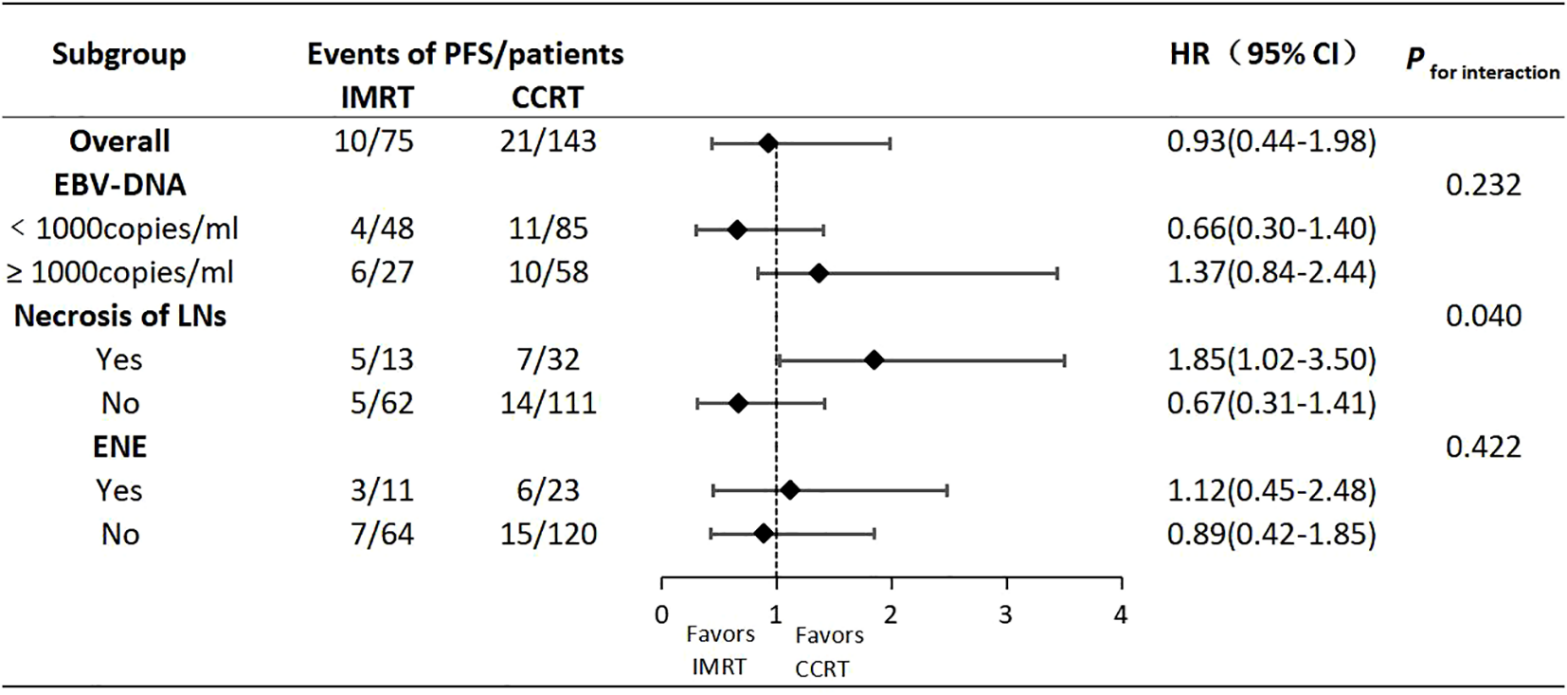
Forest plots of PFS according to subgroups.

We conducted further subgroup analyses on the three independent risk factors identified above, hoping to identify specific subpopulations that might benefit from different treatments. As shown in **Figure 4**, regarding PFS, there was an interaction effect between lymph node necrosis (yes vs. no) and treatment modality (IMRT vs. CCRT) (*P* for interaction=0.040). But the other two factors did not have an interaction with the treatment modality(*P* for interaction>0.05 for both comparisons). In the subgroup with lymph node necrosis, IMRT compared to CCRT resulted in a poorer prognosis (HR:1.85,95% CI:1.02-3.50). However, in the subgroup without lymph node necrosis, there was no significant difference in prognosis between IMRT and CCRT (HR:0.67,95% CI:0.31-1.41). This suggested that T2N1 patients with lymph node necrosis might be potential candidates for CCRT.

### Acute toxicities

As shown in **Table 3**, the incidence of acute hematological toxicity, gastrointestinal toxicity, hepatotoxicity, and nephrotoxicity was significantly higher in the CCRT group compared to the IMRT group (P<0.05 for all comparisons).

**Table 3:** Comparison of acute toxicities between the two groups of patients.

Acute toxicities	IMRT[cases(%)]		CCRT[cases(%)]		*P*
	Grade 1-2	Grade 3-4	Grade 1-2	Grade 3-4	
Hematologic toxicity	15 (20.0)	2 (2.7)	85 (59.4)	14 (9.8)	<0.001
Gastrointestinal toxicity	8 (10.7)	2 (2.7)	58 (40.6)	22 (15.4)	<0.001
Hepatotoxicity	4 (5.3)	0 (0.0)	29 (20.2)	1 (0.7)	0.010
Nephrotoxicity	4 (5.3)	0 (0.0)	41 (28.7)	1 (0.7)	<0.001
Oral mucositis	64 (85.3)	11 (14.7)	114 (79.7)	29 (20.3)	0.309

## Discussion

As previously mentioned, in the era of IMRT, the role of concurrent chemotherapy for stage II NPC remains a matter of debate. Two prospective clinical studies have shown that for stage II NPC, CCRT compared to IMRT alone had comparable efficacy but increased side effects. However, among the three subtypes of stage II NPC, the T2N1 subtype has the worst prognosis. Whether concurrent chemotherapy can be universally dismissed for this specific type of NPC requires further research. To the best of our knowledge, this study is the first to focus exclusively on the T2N1 subtype, rather than all stage II patients,to explore the value of concurrent chemotherapy on the basis of IMRT. The results showed that while CCRT compared to IMRT alone showed comparable efficacy in the overall population, certain specific subgroups might benefit more from CCRT.

During the process of case collection, we found that T2N1 patients were more likely to receive radiotherapy combined with chemotherapy, while T2N0 and T1N1 patients were more likely to receive IMRT alone. As evidenced by the final number of cases included in this study, the number of CCRT patients was nearly twice that of IMRT patients. This suggests that clinicians tend to increase treatment intensity by opting for CCRT over IMRT for the T2N1 subtype, which has the worst prognosis among stage II patients, due to concerns about treatment failure and a lack of confidence in IMRT alone. This also underscores the urgent need to further clarify the role of concurrent chemotherapy in this subtype. Finally, our study specifically focused on the T2N1 subtype to explore the value of concurrent chemotherapy on the basis of IMRT. Although the final number of patients in the two groups was not balanced, the baseline data of the two groups were comparable. Considering the sample size and the small number of survival endpoint events, the propensity score matching (PSM) method was not used for analysis (15).

Previous studies have shown that the effect of concurrent platinum-based chemotherapy during radiotherapy was related to the cumulative dose rather than the treatment regimen. Whether it was a weekly or every three weeks regimen, as long as the cumulative dose reached 200mg/m^2^ or more, the best therapeutic effect could be achieved (16). Therefore, this study’s inclusion criteria required a cumulative dose of concurrent platinum-based chemotherapy of at least 200mg/m^2^ to fully and realistically evaluate the role of concurrent chemotherapy. Lymph node size (17), necrosis (18), ENE (19) and lymph node metastasis in the level III (20) have been confirmed as poor prognostic factors for stage II NPC and were also collected as baseline data. Plasma EBV-DNA, released by tumor cells, has a pre-treatment copy level positively correlated with tumor burden, making it one of the most significant prognostic factors for NPC (21). The cutoff value of 4000 copies/ml for EBV-DNA used in the 2022 phase III clinical study (7) was derived from previous studies that included NPC patients of all stages (22). This value may not be suitable for stage II NPC and may vary across different centers due to differences in detection instruments and reagents, which warrants further exploration. In this study, a cutoff value of 1000 copies/ml for PFS was determined through ROC curve analysis. GTVnx volume,as an important prognostic factor, is more sensitive than T staging (23), and this study also determined the cut-off value of 22ml through ROC curve analysis.

Multiple studies have reported that T2 and N1 were high-risk factors for distant metastasis in stage II NPC,and the T2N1 subtype had the poorest prognosis, with the highest distant metastasis rate among the three subtypes (9–11). This study also showed that the distant metastasis rate in T2N1 was significantly higher than the locaregional recurrence rate, indicating that distant metastasis is the main mode of treatment failure. The 5-year PFS, OS, LRRFS and DMFS rates in this study were comparable to the data from previous studies on the T2N1 subtype. Additionally, there were no differences for the entire population in 5-year survivals between the IMRT and CCRT groups, consistent with previous findings on the T2N1 subtype (9–11). Univariate analysis for the primary endpoint PFS showed that EBV-DNA copy level, GTVnx volume, maximum diameter of cervical lymph nodes, lymph node necrosis and ENE were prognostic factors. Multivariate analysis ultimately identified EBV-DNA copy level, lymph node necrosis and ENE as the independent prognostic factors for PFS. Therefore, for T2N1 patients with EBV-DNA≥1000 copies/ml, lymph node necrosis or ENE, the risk of failure is increased, and CCRT may be considered as appropriate. The GTVnx volume was significant in univariate analysis but did not enter the multivariate model, possibly because the primary tumor volume of stage II patients is not large and does not involve the skull base or intracranial invasion. For IMRT planning, it is relatively easy to achieve good target dose coverage and excellent local control while attempting to minimize the dose to surrounding normal organs. Previous studies have shown that improved local control rate could also reduce the distant metastasis rate, thereby translating into survival benefits (24). Thus, the impact of GTVnx volume on the prognosis of stage II NPC is estimated to be not as significant as it is in locally advanced stages. The reason why EBV-DNA copy level entered the multivariate model and the maximum diameter of cervical lymph nodes did not may be because EBV-DNA is positively correlated with tumor burden,reflecting the total burden of the primary lesion and lymph nodes. It carries more weight and is more representative of tumor burden than the maximum diameter of cervical lymph nodes, which only represents lymph node burden. This may also explain why GTVnx volume, which only represents the burden of the primary lesion, did not enter the multivariate model. In this study, no differences in 5-year survivals were observed between the IMRT and CCRT groups in the overall T2N1 population. Subgroup analysis was conducted with the hope of identifying specific subpopulations that might benefit from different treatments. The results showed an interaction effect between lymph node necrosis (yes vs. no) and treatment modality (IMRT vs. CCRT) on PFS. In the subgroup with lymph node necrosis, IMRT compared to CCRT resulted in poorer prognosis. This suggests that T2N1 patients with lymph node necrosis may be potential candidates for CCRT, as lymph node necrosis indicates hypoxic conditions that may lead to inherent radioresistance. Concurrent platinum-based chemotherapy can enhance radiosensitivity and improve treatment efficacy (18). In terms of side effects, CCRT inevitably increased acute hematological, gastrointestinal and other toxicities compared to IMRT.

As with any retrospective study,there are several inherent limitations. Firstly, potential confounding factors related to prognosis may have biased the results. Secondly, subgroup analysis has its own drawbacks, such as reduced sample size, baseline imbalances and an increased risk of type I errors due to multiple comparisons (25). Therefore, the results of this analysis are only exploratory and should be interpreted with caution. Thirdly,due to incomplete medical records, late toxicities after radiotherapy could not be assessed. In summary, the findings of this study need further confirmation by prospective randomized clinical trials. Targeted and immunotherapies are emerging trends. Future clinical studies for stage II NPC requiring CCRT could explore the use of targeted drugs like nimotuzumab or PD-1/PD-L1 monoclonal antibodies as alternatives to platinum-based chemotherapy, aiming to reduce toxicity without compromising efficacy.

In summary,this study contributes valuable insights into treatment decision-making for patients with this specific type of NPC. For the entire population of T2N1 subtype of NPC, the therapeutic effects of IMRT and CCRT are comparable, with increased acute hematological, gastrointestinal and other toxicities in the latter. However, for patients with EBV-DNA copy level ≥1000 copies/ml, lymph node necrosis and ENE, the risk of failure is increased, and CCRT may be considered as appropriate. In particular, patients with lymph node necrosis may be potential beneficiaries of CCRT, warranting further investigation.

## Data Availability

The original contributions presented in the study are included in the article/supplementary material. Further inquiries can be directed to the corresponding author.
